# The Relationship Between Muscle Strength and Cognitive Performance Across Alzheimer's Disease Clinical Continuum

**DOI:** 10.3389/fneur.2022.833087

**Published:** 2022-05-12

**Authors:** Marco Filardi, Roberta Barone, Giulia Bramato, Salvatore Nigro, Benedetta Tafuri, Maria Elisa Frisullo, Chiara Zecca, Rosanna Tortelli, Giancarlo Logroscino

**Affiliations:** ^1^Department of Basic Medicine, Neuroscience and Sense Organs, University of Bari “Aldo Moro,” Bari, Italy; ^2^Center for Neurodegenerative Diseases and the Aging Brain, University of Bari “Aldo Moro” at Pia Fondazione “Card. G. Panico”, Tricase, Italy; ^3^Department of Geriatrics, Fondazione Policlinico Universitario A. Gemelli IRCCS, Università Cattolica Sacro Cuore, Rome, Italy; ^4^Institute of Nanotechnology (NANOTEC), National Research Council, Lecce, Italy

**Keywords:** mild cognitive impairment, Alzheimer's disease, handgrip strength, attention, working memory, declarative memory

## Abstract

Alzheimer's disease (AD) is a neurodegenerative disorder characterized by a progressive cognitive decline, mostly prominent in the domain of memory, but also associated with other cognitive deficits and non-cognitive symptoms. Reduced muscle strength is common in AD. However, the current understanding of its relationship with cognitive decline is limited. This study investigates the relationship between muscle strength and cognition in patients with AD and mild cognitive impairment (MCI). We enrolled 148 consecutive subjects, including 74 patients with probable AD dementia, 37 MCI, and 37 controls. Participants underwent neuropsychological evaluation focused on attention, working memory, declarative memory and learning. Muscle strength and muscle mass were measured through hand dynamometer and bio-electrical impedance analysis, respectively. Patients with AD dementia were divided with respect to the severity of cognitive impairment into mild and moderate-to-severe patients. Moderate-to-severe patients with AD presented lower handgrip strength than MCI and controls. No differences were observed in muscle mass. In MCI and AD dementia, handgrip strength was associated with overall cognitive functioning, attentional and memory performance. The routine implementation of handgrip strength assessment in the clinical work-up of patients with MCI and AD could potentially represent a simple method to monitor functional and cognitive decline along the disease course.

## Introduction

Alzheimer's disease (AD) is the most frequent cause of cognitive impairment and dementia in elderly people. For the number of subjects involved and the cost of health care, it is considered a major global public health challenge (1). AD is characterized by a gradual and progressive cognitive decline, usually starting in the domain of memory and accompanied by the involvement of other cognitive domains and, at various degrees, behavioral disturbances (2). Historically AD has been considered a clinico-pathophysiological entity with discrete clinical stages, namely mild cognitive impairment (MCI) and dementia (3).

However, during the last decade, the growing interest in preclinical disease stages (4), coupled with the development of reliable disease biomarkers (5), has prompted a shift toward a new disease model. AD is currently considered a multifaceted process along a cognitive and biological continuum going from preclinical (cognitively unimpaired individuals with biochemical evidence of AD pathology) to clinical stages [MCI due to AD and AD dementia (6)].

Along with the typical neuropsychological and behavioral disturbances, patients with AD frequently exhibit non-cognitive symptoms such as sleep and circadian rhythm disruption (7), fatigue (8), balance dysfunction (9), and decline in upper and lower extremity motor performance (10). Non-cognitive symptoms are prominent in the later AD stages, but they can occur early in the disease course and even precede of years the onset of cognitive symptoms, thus representing a potential predictor of subsequent cognitive decline (11). Handgrip strength (HGS) is a reliable and relatively simple method to assess total upper extremity muscle strength (12). HGS is considered among the main indicators of overall physical status and has been proposed as a useful tool for monitoring the trajectory of cognitive decline in the elderly (13). On the other hand, cross-sectional studies that investigated the relationship between HGS and cognition in elderly non-demented individuals showed that reduced HGS is associated with poorer cognitive functioning and lower performance in the domains of attention, memory, and executive functions (14, 15). Nevertheless, the current understanding of the relationship between reduction of muscle strength and cognitive deficits in patients with AD is still limited, with the few available studies specifically focused on assessing differences in muscle strength with respect to AD clinical stages (16), and on exploring the relationship between HGS and overall cognitive functioning in patients with AD dementia and MCI (17). The present study aims to investigate: (a) the cross-sectional differences in muscle strength and muscle mass in patients across the AD spectrum and (b) the relationship between HGS and cognitive performance in patients with AD and MCI, with a specific focus on the cognitive domains that are more compromised in AD, namely memory and attention.

## Materials and Methods

### Participants

The study includes 148 consecutive subjects referred, between January 2019 and January 2020, for memory complaints to the Center for Neurodegenerative Diseases and the Aging Brain of the University of Bari Aldo Moro in Tricase with a final diagnosis of MCI or probable AD dementia according to the National Institute on Aging and Alzheimer's Association (NIA-AA) diagnostic guidelines (18, 19).

All subjects underwent a diagnostic protocol encompassing (a) clinical evaluation performed by a neurologist specialized in neurodegenerative disorders; (b) comprehensive neuropsychological assessment (cognitive domains investigated: attention, visuospatial abilities, memory, executive functions and language); (c) high-field (3-Tesla) brain magnetic resonance imaging (MRI) scan and, whenever possible; (d) lumbar puncture to quantify cerebrospinal fluid AD biomarkers.

The final sample consists of 37 MCI patients (i.e., subjects presenting with modest impairment in one or more cognitive domains at neuropsychological assessment without significant interference with basic activities of daily living) and 74 patients who met the diagnostic criteria for probable AD dementia (i.e., subjects presenting with a significant decline of memory, learning and of at least one other cognitive domain and loss of independence in performing everyday life activities).

Lumbar puncture was performed in 42/74 probable patients with AD, all presenting evidence of AD pathology based on the core AD biomarkers framework (5).

The control group consisted of 37 subjects who underwent the above-mentioned diagnostic assessment without objective evidence of cognitive impairments and MRI abnormalities. The study was conducted in accordance with the Declaration of Helsinki and approved by the local health trust's ethics committee (ASL Lecce verbale n°6, 25 July 2017). Written informed consent was obtained from all participants.

### Muscle Strength and Muscle Mass Assessment

Height and weight were measured for each subject to calculate body mass index (BMI) (weight in kg/height in meters squared, kg/m^2^). According to World Health Organization's criteria, participants were stratified based on BMI values as underweight (BMI <18.5), normal weight (BMI: 18.5–24.9), overweight (BMI: 25.0–29.9), and obese (BMI≥30). Body composition was assessed via bio-electrical impedance analysis (BIA), which quantifies body composition measuring the electrical resistance properties of tissues. Bioelectrical resistance was evaluated through BIA101 Anniversary (Akern s.r.l., Montacchiello, Italy) at frequencies of 50 kHz. All the measures were performed by the same expert nutritionist in a room with controlled temperature (24–26°C) with the patients laying in supine position and electrodes placed on the right hand and foot. The skeletal muscle mass index (SMI), computed as skeletal muscle mass (in kg) divided by height (in m) squared, was considered as a measure of total muscle mass. Upper extremity muscle strength was measured using the DynEx hand dynamometer (Akern s.r.l., Montacchiello, Italy). Grip strength was assessed twice for each hand and reported as the average of maximum handgrip strength (in kg).

### Neuropsychological Assessment

Different memory functions (i.e., working memory, immediate recall, delayed recall, and learning), attention, and an overall measure of cognitive functioning were considered.

The digit span was administered as a measure of attention and working memory (20). The task involves two conditions, both requiring the repetition of a series of digits with increasing difficulty, where in the forward condition (attention) the digits must be repeated in the same order as presented and in the backward condition (working memory) the digits must be repeated in reverse order. The Rey Auditory Verbal Learning Test (RAVLT) was administered as a measure of declarative memory and verbal learning (21). A list of 15 unrelated words is presented and the subjects have to recall as many words as possible (immediate recall). After five repetitions a second list is presented and, similarly, the subjects have to recall as many words as possible. Thereafter subjects have to recall words from the first list (delayed recall). Finally, a list of 50 words is presented (containing words from both lists and 20 novel words) and subjects have to recognize if the words belong to the first and second list presented (recognition). The Mini-Mental State Examination (MMSE) was administered as a global measure of cognitive functioning (22). Activities of daily living (ADL) and instrumental activities of daily living (IADL) were also investigated thought validated scales (23, 24).

### Statistical Analysis

Data were explored with descriptive statistics (mean ± standard deviation or frequency). For statistical purpose, patients with AD dementia were classified either as “mild” and “moderate-to-severe” according to MMSE score [between 21–25 and ≤ 20, respectively (25)].

Group differences in the demographic and anthropometric data were analyzed with chi-square test and one-way analysis of variance (ANOVA) followed by Bonferroni *post-hoc* test. Analysis of covariance (ANCOVA) was conducted to analyze groups' differences in HGS, SMI, and neuropsychological data while controlling for age and gender. Bonferroni *post-hoc* test was used to assess between-group differences in estimated marginal means. The Benjamini–Hochberg false discovery rate (FDR) procedure was used to correct for multiple comparisons (26). The relationship between HGS, age, SMI, BMI, and neuropsychological measures (digit span forward/backward and RAVLT immediate/delayed recall and recognition) were explored, separately for cases and controls, through Spearman correlation coefficient analysis. Statistical analyses were conducted using R (27) and SPSS 19.0 (SPSS, Inc. Chicago, IL). Results with FDR adjusted *p* < 0.05 were considered statistically significant.

## Results

### Muscle Strength and Muscle Mass

Demographic and clinical variables of the whole sample and stratified by clinical stages are reported in Table 1. No group differences were observed in gender, BMI, and BMI category distribution. Significant differences emerged in age with both mild AD and moderate-to-severe patients with AD being significantly older than controls (*p* < 0.05 and *p* < 0.0005, respectively).

**Table 1:** Demographic, clinical characteristics, and neuropsychological data.

	**Controls**	**MCI**	**Mild AD**	**Moderate-severe AD**	**F_**(3, 142)**_**	**FDR-adjusted *p*-value**
	**(*n* = 37)**	**(*n* = 37)**	**(*n* = 20)**	**(*n* = 54)**		
	**Mean ±SD**	**Mean ±SD**	**Mean ±SD**	**Mean ±SD**		
*Demographic and clinical data*						
Male/female	16/21	19/18	10/10	20/34	2.18	*ns*
Age, *y*	63.43 ± 11.64	68.19 ± 7.76	71.40 ± 9.78	72.06 ± 8.32	6.84	<0.0005
BMI	27.80 ± 4.71	26.89 ± 4.31	26.01 ± 3.17	26.07 ± 3.80	1.53	*ns*
BMI category (under\ normal\ over\obese)	1/10/14/12	0/11/21/5	0/8/10/2	1/22/23/8	10.30	*ns*
*Muscle strength and mass*						
HGS, *kg*	22.46 ± 6.20	22.19 ± 6.92	18.79 ± 5.19	17.32 ± 6.74	5.65*	<0.001
SMI	9.86 ± 1.52	9.62 ± 1.96	9.76 ± 1.60	9.31 ± 1.39	0.32*	*ns*
*Neuropsychological data*						
Digit span, forward	5.62 ± 0.92	5.30 ± 0.91	5.15 ± 0.88	4.41 ± 1	11.94*	<0.0001
Digit span, backwards	4.05 ± 0.85	3.41 ± 0.96	3.20 ± 1.06	2.24 ± 1.01	24.70*	<0.0001
RAVLT, immediate recall	36.51 ± 8.90	26.16 ± 7.16	19.16 ± 5.43	14.17 ± 6.59	57.48*	<0.0001
RAVLT, delayed recall	6.05 ± 2.55	3.86 ± 2.67	1.20 ± 1.58	0.46 ± 1.11	48.87*	<0.0001
RAVLT, recognition	0.94 ± 0.24	0.83 ± 0.22	0.65 ± 0.19	0.66 ± 0.15	10.57*	<0.0001
MMSE	27.51 ± 1.97	26.03 ± 1.50	21.80 ± 0.77	15.26 ± 3.64	192.35*	<0.0001
*Activities of daily living*						
ADL	0.10 ± 0.31	0.14 ± 0.35	0.45 ± 0.83	0.58 ± 1.13	2.14*	*ns*
IADL	0.15 ± 0.37	0.69 ± 1	1.35 ± 1.93	3.15 ± 2.23	19.37*	<0.0001

MCI, mild cognitive impairment; AD, Alzheimer's disease; FDR, false discovery rate; BMI, body mass index, HGS, handgrip strength; SMI, skeletal muscle index, RAVLT, rey auditory verbal learning test; MMSE, mini mental state examination; ADL, activities of daily living; IADL, instrumental activities of daily living; ns, not significant. ^*^ANCOVA corrected for age and gender.

The ANCOVA showed statistically significant group differences for HGS but not in SMI.

*Post-hoc* analysis showed that moderate-to-severe AD present lower HGS than MCI (17.32 ± 6.74 vs. 22.19 ± 6.92, *p* < 0.05) and controls (17.32 ± 6.74 vs 22.46 ± 6.20, *p* < 0.005).

### Neuropsychological Data

Significant differences emerged in the forward digit span, with moderate-to-severe AD reporting fewer digits than controls (*p* < 0.0001) and patients with MCI (*p* < 0.0005) and mild AD (*p* < 0.05). Difference also emerged in the backward digit span with moderate-to-severe AD reporting fewer digits than controls (*p* < 0.0001) and patients with MCI (*p* < 0.0001) and mild AD (*p* < 0.005). Patients with MCI and mild AD reported fewer digits than controls (*p* < 0.05 and *p* < 0.01, respectively). Significant differences emerged in RAVLT immediate recall with moderate-to-severe AD presenting lower scores than MCI (*p* < 0.0001) and controls (*p* < 0.0001), mild AD presenting lower scores than MCI (*p* < 0.05) and controls (*p* < 0.0001), and MCI presenting lower scores than controls (*p* < 0.0001). The same trend of differences was observed for RAVLT delayed recall (all *p* < 0.001) and for RAVLT recognition score (all *p* < 0.05), although for the latter no differences emerged between MCI and controls. MMSE did not differ between controls and patients with MCI, with both presenting higher MMSE than patients with mild and moderate-to-severe AD (all *p* < 0.0001). Finally, no group differences emerged in ADL but moderate-to-severe AD presented a higher number of IADL limitations than mild AD, MCI and controls (all *p* < 0.005).

### Correlation Analysis

**Figure 1:**
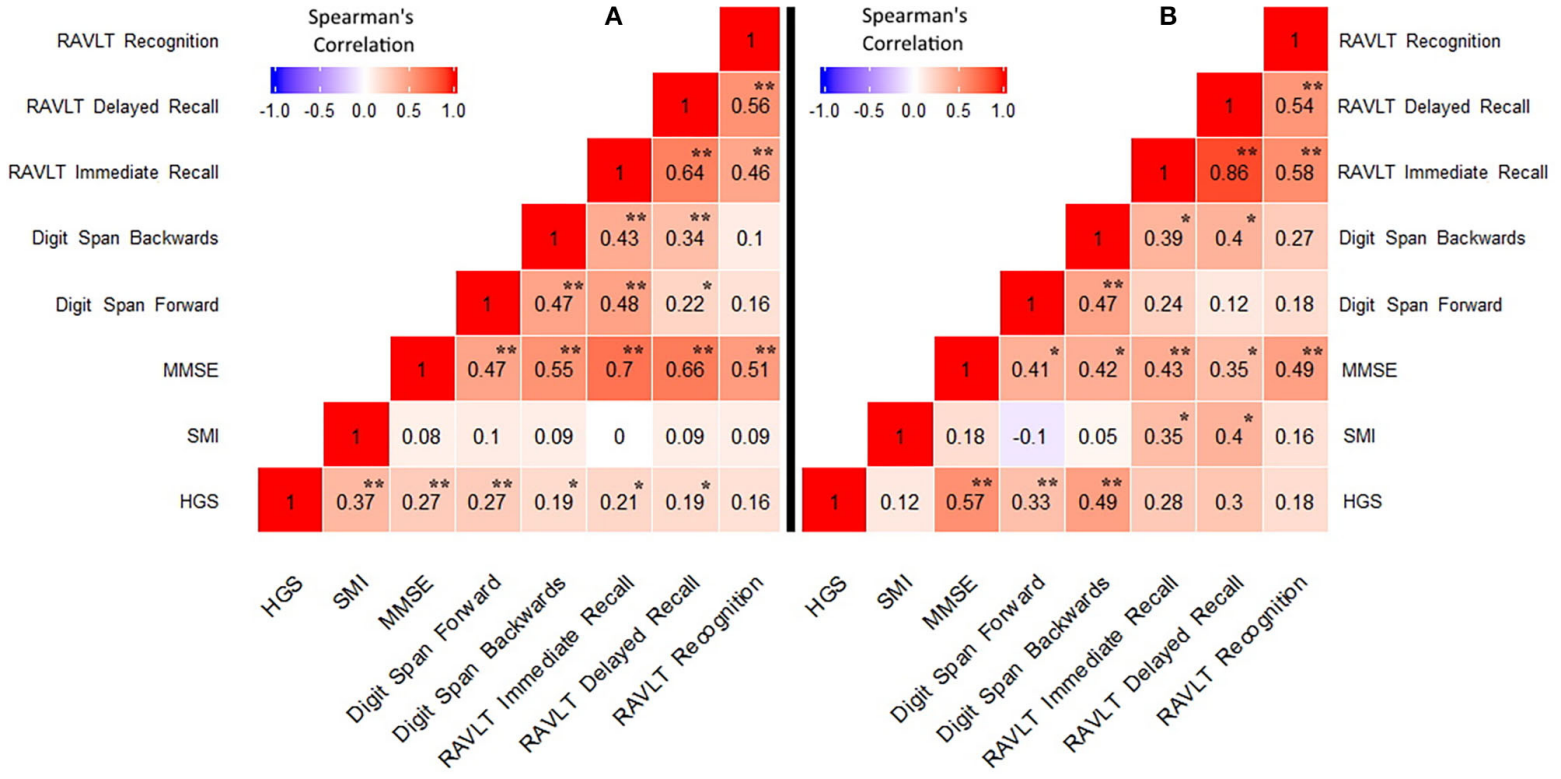
Correlations between handgrip strength, skeletal muscle index, and neuropsychological data. **(A)** MCI/AD dementia group; **(B)** Control group. HGS, Handgrip strength; SMI, skeletal muscle index; MMSE, Mini Mental State Examination; RAVLT, Rey Auditory Verbal Learning Test. *p < 0.05, **p < 0.005.

Heatmaps of Spearman correlation matrix are reported in Figure 1.

**Figure 2:**
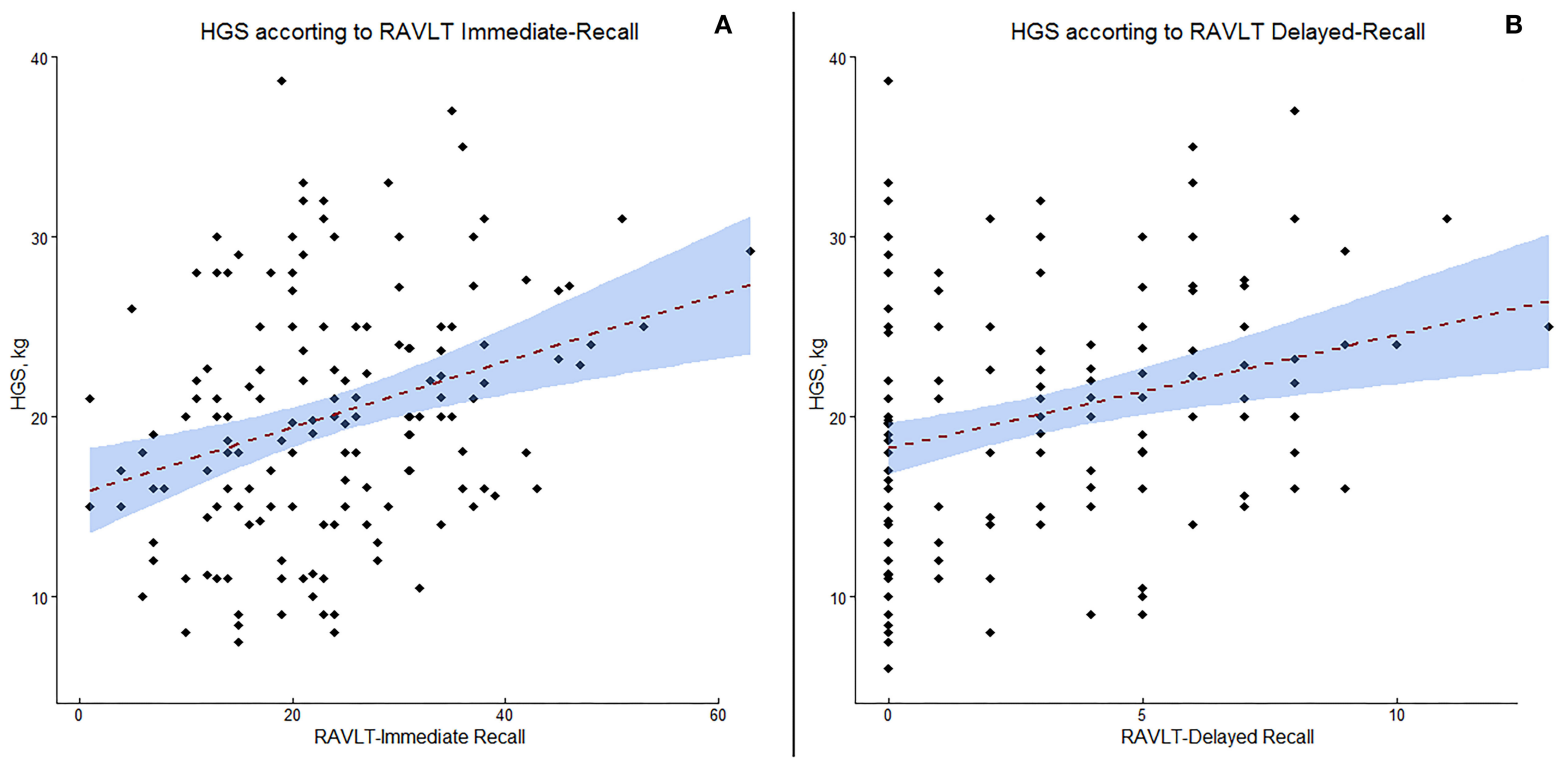
Scatter plot of the correlation between handgrip strength (HGS) and Rey Auditory Verbal Learning Test (RAVLT) immediate recall **(A)** and delayed recall **(B)**.

In the cases a) HGS was directly related to SMI (*p* < 0.0001); b) HGS was positively correlated with MMSE score (*p* < 0.005); c) HGS was positively correlated with digit span forward (*p* < 0.005) and backwards (*p* < 0.05); d) HGS was positively correlated with RAVLT immediate and delayed recall (*p* < 0.05); and e) HGS was negatively correlate with IADL (*p* < 0.005). No significant relationship emerged between SMI and neuropsychological data. In controls, a positive correlation emerged between HGS and MMSE (*p* < 0.0005), digit span forward (*p* < 0.05) and backwards (*p* < 0.005), while HGS resulted negatively correlated with ADL and IADL (all *p* < 0.05). Finally, a positive relationship emerged between SMI and RAVLT immediate and delayed recall (*p* < 0.05). A scatter plot of the relationship between HGS and RAVLT immediate and delayed recall is presented in Figure 2.

## Discussion

In this study we investigated cross-sectional differences in muscle strength and muscle mass and the relationship between muscle strength and cognitive performance in a sample of patients along the clinical continuum of AD.

After controlling for relevant covariates, we found that handgrip strength is reduced in patients with moderate-to-severe AD compared to MCI and controls, and it is linked to overall cognitive functioning and performance at tasks assessing attention and memory in both controls and patients with MCI/AD dementia. Overall, our results are in line with previous studies that documented a reduction of HGS in later stages of AD and disclosed an association between HGS and cognitive performance.

Ogawa et al. assessed differences in muscle strength and muscle mass between patients with different stages of AD (namely early, mild, and moderate AD) and found a reduction of handgrip strength in patients with moderate-stage AD compared to controls and patients with early-stage AD, and in patients with mild and early-stage AD compared to controls [MCI patients were not included in this study (16)].

Furthermore, in contrast with our results, they have also observed a reduction of muscle mass (SMI) in patients with moderate AD compared to controls and to all other AD stages. Similarly, Su et al. found reduced handgrip strength in patients with AD compared to controls, as well as in MCI patients compared to controls (17).

Age-related loss of muscle strength, not attributable to neurological or medical conditions, is called dynapenia and is highly associated with both mortality and physical disability (28).

In the elderly without dementia, it has been shown that the reduction in muscle strength is independent of the reduction in muscle mass since these two conditions show different trajectories of decline (29, 30). Accordingly, we did not observe an association between muscle strength and muscle mass in elderly controls, but we did disclose a significant association among patients.

Concerning the association between handgrip strength and neuropsychological performance in elderly non-demented individuals, our results support the large corpus of scientific literature that disclosed an association between muscle strength and different aspects of cognitive functioning. Several studies have firmly established a link between a reduction of handgrip strength and poorer global cognitive functioning [assessed through the MMSE and the Montreal Cognitive Assessment, MoCA; (14, 31)], or lower performance in specific cognitive domains most notably attention, executive functions (32), and different aspect of memory functioning (33).

By contrast, studies that investigated the relationship between handgrip strength and cognition in patients with AD are rare and have generally explored this relationship focusing on overall cognitive functioning only. A very recent study by Su et al. revealed a positive relationship between HGS and global measures of cognitive functioning [i.e., MMSE, MoCa and Addenbrooke's Cognitive Examination (17)].

Our results confirm and extend these findings by showing that, in patients along the AD continuum, higher HGS is associated with better global cognitive functioning and with higher performance in the neuropsychological domains most affected in AD dementia, namely declarative memory, working memory, and attention (34).

The mechanisms underlying the relationship between decreased HGS and cognitive impairment are yet to be completely elucidated. It has been hypothesized that muscle strength and cognition might share the same brain regions and networks (35), and that the neuroanatomical and neurochemical changes occurring with physiological brain aging could represent one of factors underpinning this relationship (36).

Evidence in support of this hypothesis arise from interventional studies on elderly non-dement individuals who showed a positive effect of resistance and strength training on cognitive functioning (37, 38). Moreover, subsequent studies have hinted a possible mechanism through which strength training improves cognition by showing that exercise-related cognitive improvement is mediated by a reduction in white matter degradation and changes in hemodynamic activity across several brain areas (39, 40). When considered as a whole, our results suggest that HGS can be considered a reliable indicator of cognitive and functional decline in later-stage patients with AD.

These finding could have important clinical implications in the clinical work-up and in the follow-up of patients with MCI and AD. Indeed, to date there is no disease-modifying therapy for AD and the available pharmacological treatments, mainly aimed to mitigate memory loss and improve daytime functioning, show limited efficacy (41). On the other hand, randomized controlled trials of muscle strength training and multi-domain lifestyle interventions showed beneficial effects on cognitive functioning in elderly subjects at a high risk of developing dementia (42). In this scenario, HGS could represent a promising endpoint to assess the effectiveness of such interventions. Moreover, bearing in mind that HGS is a simple, reliable, and inexpensive measure, it could be implemented in the routine outpatient clinical evaluation of patients with AD and MCI, as a method potentially able to provide useful information on disease course.

Some limitations of the present study should be acknowledged.

First, the sample size is relatively small, although sufficient to detect meaningful statistical difference and in line with those of several single-center study.

Second, MCI and AD diagnosis was clinical and not biomarker-based as lumbar puncture was available in only a little over half of patients with AD and not available in patients with MCI, therefore the clinical sample may be heterogeneous.

Third, we focused exclusively on HGS and did not consider other important indicators of motor performance (i.e., lower extremity strength, gait speed, and balance).

To conclude, our study showed that HGS is reduced in patients with later stage AD and is associated with overall worse cognitive performance and attentional and memory impairments. Further studies are needed to explore whether HGS could represent a reliable marker of functional and cognitive decline along the AD disease course.

## Data Availability Statement

The raw data supporting the conclusions of this article will be made available by the corresponding author, upon reasonable request.

## Ethics Statement

The studies involving human participants were reviewed and approved by ASL Lecce verbale n°6, July 25th, 2017. The patients/participants provided their written informed consent to participate in this study.

## Author Contributions

MFi, RT, and GL: conceptualization. MFi, RB, SN, and CZ: methodology. MFi and BT: formal analysis. RB, GB, MFr, and RT: investigation. RB, GB, SN, and CZ: data curation. MFi, RB, and RT: writing—original draft preparation. MFi, RB, GB, SN, BT, MFr, CZ, RT, and GL: writing—review and editing. RT and GL: supervision. GL: funding acquisition. All authors have read the manuscript and approved it for publication.

## Funding

This research was funded by Regione Puglia and CNR for Costituzione del Tecnopolo per la Medicina di Precisione. D.G.R. n. 2117 of 21.11.2018.

## Conflict of Interest

RT is a full-time employee of F. Hoffmann-La Roche, Ltd., outside of the submitted work. GL reports personal fees from Roche and Amplifon, outside of the submitted work. The remaining authors declare that the research was conducted in the absence of any commercial or financial relationships that could be construed as a potential conflict of interest.

## Publisher's Note

All claims expressed in this article are solely those of the authors and do not necessarily represent those of their affiliated organizations, or those of the publisher, the editors and the reviewers. Any product that may be evaluated in this article, or claim that may be made by its manufacturer, is not guaranteed or endorsed by the publisher.
